# Blood Pressure Targets: Will We Reach Definite Figures? I Currently Have Mine

**DOI:** 10.5935/abc.20170100

**Published:** 2017-07

**Authors:** Paulo César B. Veiga Jardim

**Affiliations:** Faculdade de Medicina Universidade Federal de Goiás (UFG); Pós-graduação em Ciências da Saúde e Nutrição em Saúde da UFG; Liga de Hipertensão Arterial da UFG, Goiânia, GO - Brazil

**Keywords:** Hypertension, Blood Pressure / physiology, Antihypertensive Agents, Diabetes Mellitus, Blood Pressure Targets

I was born before the era of evidence-based Medicine. When I was a student, arterial hypertension (AH) used to be diagnosed based on blood pressure (BP) readings greater than 160/95 mm Hg.

Can anyone imagine that now?

Cardiology has advanced a lot in all directions, both in diagnostic and treatment methods. Currently, treatment is based on scientific evidence, and the drug armamentarium available is extremely effective.

But let us go back to AH.

Over the years, we have learned through a large number of studies, initially observational ones, and then intervention ones, that, if on the one side cardiovascular risk increases with BP levels from 115/75 mm Hg on, doubling with every 20-mmHg increase in systolic BP (SBP) and 10-mmHg increase in diastolic BP (DBP), on the other, cardiovascular risk decreases significantly with BP reduction by using the pharmacological treatment offered to hypertensives.^1-5^

There is no definite evidence that non-pharmacological treatment (healthy lifestyle) yields the same results, but that is an obvious universally accepted assumption, although difficult to implement nowadays.^1-5^

Excellent antihypertensive agents have been developed and perfected. The benefits of their use regarding both morbidity and mortality have been proven.^1-5^

There is no doubt about that.

We face, however, some dilemmas, beginning with access to healthcare services and medications, when required. That is crucial, depends on consistent public policies that change the *status quo*; nevertheless, that is not the object of this discussion.^1-5^

Another aspect, concerning treatment itself, is the huge challenge posed by adherence to treatment. Currently, a small number of individuals, aware of their hypertensive condition and of the risks inherent to it, and even having access to the healthcare system, do not adhere to the proposed treatment. This critical issue involves educational measures as the major tool for that behavior change, which is common and universal, but, once again, this is not the object of my observations today.^1-5^

Moving on to what matters!

In the past 40 years, hundreds of studies on the treatment of AH were conducted worldwide, providing us with the opportunity to find that pharmacological therapy modifies the natural history of AH, significantly reducing cardiovascular mortality and morbidity. It is worth noting that this fact is rather due mainly to BP reduction, than to the type of drug used.

Therefore, the epidemiological cycle is closed: BP reduction is effectively beneficial.^1-5^

But how much should it be reduced? To which extent is it safe?

The dilemma of targets!

Most studies showing a reduction in cardiovascular events have been designed to compare BP levels before and after treatment. At first, active drugs were compared to placebo, assessing the changes in BP and its possible benefits. Later, active drugs have been compared to each other, aiming at finding differences between them.

The major focus was not the BP reached, but the difference between initial and final levels.

The best source of evidence in medicine is known to come from randomized controlled clinical trials. Establishing objective targets required the investigation design to have that purpose.

The HOT (*Hypertension Optimal Treatment*) study,^6^ published in 1998, was a pioneer in regard to targets, DBP being the reference used. Over 18500 patients aged between 50 and 80 years (mean, 61.5 years), with DBP between 100 and 115 mmHg, were allocated to DBP targets of ≤ 90 mm Hg, ≤ 85 mmHg and ≤ 80 mmHg. As DBP levels lowered, greater reductions in cardiovascular events were observed, the greater benefit occurring in the group of patients whose DBP was reduced to the mean level of 82.6 mm Hg. Reductions to levels below those were observed to be safe, and, among patients with diabetes, the benefits were even higher for the group whose target was DBP ≤ 80 mmHg. That study was a landmark regarding BP targets, and international guidelines have based their recommendation on it for years.^1-4,6^

Over time, there have been hundreds of good quality investigations on drug interventions, involving thousands of patients, and they have continued to support the benefits of BP control; however, the ones assessing targets have been scarce.^1-4,6^

Many might be thinking on some studies aimed at establishing some targets, such as the Italian Cardio-Sis, published in 2009, that investigated non-diabetic hypertensives over the age of 55 years, and aimed at SBP targets < 130 mmHg or < 140 mmHg. In that study, the intermediate primary outcome ‘left ventricular hypertrophy’ showed significantly favorable results for lower BP levels, and positive results regarding pre-specified secondary outcomes, which were cardiovascular.^7^

Similarly, in 2008, the CASE-J trial, involving only elderly individuals, was published reporting significant advantages for lower BP levels. In addition, in 2008, the JATOS study, assessing elderly, showed no difference between SBP targets under 140 mm Hg or 160 mm Hg.^8,9^

Until then, all official documents, including our last guideline, assessing what BP levels should be pursued for greater benefits, had worked with BP under 140/90 mmHg for the general population, and under 130/80 mmHg for individuals at high cardiovascular risk, those with cardiovascular disease, diabetes and established kidney disease.^1-4,10,11^

In 2010, the result of the ACCORD study was published.^12^ It assessed only patients with diabetes, and tried to define if stricter BP control targets (SBP < 120 mmHg) was advantageous over conventional targets (SBP < 140 mmHg). That study randomized more than 4500 diabetic hypertensives, with a mean age of 62 years, followed up for a mean period of 4.7 years. The primary composite outcome was non-fatal myocardial infarction, non-fatal stroke or cardiovascular death. In addition, several secondary cardiocirculatory outcomes were predefined. The results were null, that is, there was no difference in major, fatal and non-fatal cardiovascular combined events. Regarding secondary outcomes, there was no significant difference, except for fatal and non-fatal stroke, which showed a significant reduction of 41% and 37%, respectively. The group with a more marked BP reduction had more adverse events, but without jeopardizing the end of the study.^12^

That study had a huge repercussion, leading to a new rationale, which, in my opinion, was mistaken.^5^ It was implied, almost immediately, that stricter BP targets for diabetic patients would be harmful, and, indirectly there was controversy about lower BP targets for all types of patients.^5^

I assess the ACCORD study findings and interpret its results differently. The primary outcomes were similar, that is, there was no difference.^12^ I emphasize the significance of that: there was neither reduction, nor increase in primary events.^12^

On the other hand, but not less important, assessing the secondary outcome ‘stroke’, we observe that there were benefits for the group with stricter BP control.^12^ Who does not want to protect a diabetic patient from a stroke? Just food for thought!

In the sequence of the investigations for more adequate BP levels, by the end of 2015, the SPRINT study was published.^13^ Financed by the National Institutes of Health (NIH), without any conflict of interest, the protocol was carefully designed specifically aimed at assessing the more beneficial BP levels in terms of cardiocirculatory outcomes for non-diabetic hypertensives. Individuals aged 50 years or older, with SBP between 130 and 180 mm Hg and at increased cardiovascular risk were selected. For individuals randomized for intensive treatment, a SBP target < 120 mmHg was defined, while for those randomized for standard treatment, a SBP target < 140 mmHg was defined. The primary composite outcome comprised acute myocardial infarction, other acute coronary syndromes, stroke, heart failure and death from cardiovascular causes. The secondary outcomes were defined as the individual components of primary outcome, all-cause death and the addition of primary outcome or all-cause death.^13^

That study randomly assigned 9361 patients, more than 4650 to each treatment type. The mean age was 67.9 years, and the groups were homogeneous in all aspects. In the first year of follow-up, the mean SBP and DBP achieved were, respectively, 121.4 and 68.7 mm Hg in the intensive-treatment group, and 136.2 and 76.3 mm Hg in the standard-treatment group. At the end of the study, the mean SBP levels were 121.5 and 134.6 mm Hg in the intensive-treatment and standard-treatment groups, respectively.^13^

The SPRINT study, planned to last 5 years, was interrupted by the Safety Committee at 3.26 years, because a significant difference in outcomes was observed between the groups on two pre-established occasions for such control. The intensive-treatment group showed a 25%-reduction in primary outcomes, and that difference began to appear from the first year of intervention on. The number of all-cause deaths was also 27% lower in that group, in which there were 43% less deaths from cardiovascular causes. Adverse events were more frequent in that group with stricter BP control, however without significant hindrance to the ongoing research.^13^

That clinical trial was outstanding, had an excellent design, clear objectives and proper development. Its results were categorical. However, as any protocol, it is liable to be challenged, although, in that particular case, most lacked consistency.

One point raised was its early interruption.

But, how not to do that?

It was a matter of safety. How can we not offer the best to our patients? In addition, on a critical review of the study, its results cannot be challenged for that reason. In fact, the trial effectively provided valuable information on the BP targets to be pursued.

A good alternative to randomized clinical trials to confirm scientific evidence is provided by meta-analyses and systematic reviews. However, their results can be challenged, depending on the criteria used for selecting the studies to be assessed and those that should be eventually included.

Regarding BP targets, there is a reasonable number of meta-analyses and systematic reviews on the subject.

Four recent studies on that subject are worth noting: the first dates from 2015, assessing type 2 diabetic patients; two were from 2016, one by Xie et al.^16^ and the other by Ettehad et al.,^15^ both treating hypertensives in general; and the fourth, published in 2017 in the JACC, approached the same question in elderly patients.^17^ All studies showed the benefits of stricter BP control to reduce cardiovascular morbidity and mortality in those patients.^14-17^

**Figure 1:**
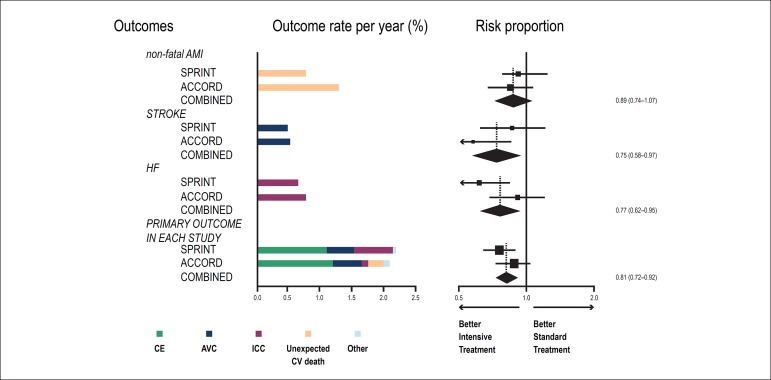
Combination of the outcomes of the SPRINT and ACCORD studies, and combined data of both studies. AMI: acute myocardial infarction; CE: coronary events; HF: heart failure; CV: cardiovascular. Adapted from Perkovic e Rodgers.^18^

In addition, it is worth emphasizing the interesting editorial by Perkovic and Rodgers^18^ published in the NEJM in November 2015, concomitantly with the SPRINT study publication. In that editorial, the authors assessed the ACCORD and SPRINT studies together, identified their similarities and differences, and understood them as complementary, creating a new situation, with an even larger number of patients, and suggested additional results. Those authors reported that, although both studies had the same BP target and similar outcomes, the ACCORD study had a smaller statistical power and primary outcomes less-sensitive to BP changes as compared to those of the SPRINT study (Figure 1). Those authors have not objectively suggested BP levels, but indicated, based on their observations and convictions, that stricter BP targets are welcome, mainly for individuals at higher cardiovascular risk.

At the beginning of 2017, Chobanian published in the JAMA a viewpoint that coincides with my understanding.^19^ Its rationale is logical and based on the existing evidence. That author suggested even stricter targets (BP < 120/80 mmHg) for individuals under the age of 50 years. He values DBP for that age group, emphasizing the concept of the importance of DBP for youngsters. He suggested BP levels below 130 mmHg for individuals aged between 50 and 74 years at high cardiovascular risk or with established disease, including those with diabetes, considering the benefits reported in the ACCORD study regarding stroke. Finally, he recommended BP levels <140 mmHg for all patients aged 75 years or older.^19^

Even considering the lack of definitive information on BP levels to be pursued in patients at high cardiovascular risk, inferring that excessively low BP levels can be harmful,^20^ those are the figures I have been working with, taking each patient’s characteristics into consideration.

Finally, it is worth noting that most individuals maintain BP levels far above any established BP target, and accepting higher BP levels is harmful, and will represent over the years a significant recrudescence of cardiovascular diseases, currently a major cause of morbidity and mortality.
